# Investing in equitable healthy aging: Why Africa must reform social pension schemes to improve Alzheimer's disease and dementia outcomes

**DOI:** 10.1002/alz.14527

**Published:** 2025-01-27

**Authors:** Cyprian M. Mostert, Najat EL Mekkaoui, Shehzad Ali, Dominic Trepel, Kirti Ranchod, Chinedu Udeh‐Momoh, Olivera Nesic, Karen Blackmon, Mary Karanja, Thomas Thesen, David Andai, Rym Ayadi, Harris Eyre, Zul Merali

**Affiliations:** ^1^ Department of Population Health Aga Khan University Nairobi Kenya; ^2^ Brain and Mind Institute Aga Khan University Nairobi Kenya; ^3^ Global Brain Health Institute, The University of Dublin, Trinity College Dublin, D01 Ireland Dublin Ireland; ^4^ University Paris Dauphine Paris France; ^5^ Western University Ontario Canada; ^6^ University of the Witwatersrand Johannesburg South Africa; ^7^ School of Medicine Wake Forest University Winston‐Salem North Carolina USA; ^8^ Euro‐Mediterranean Economists Association Barcelona Catalonia Spain; ^9^ Rice University Houston Texas USA

**Keywords:** Alzheimer's and dementia, Africa, elderly care, healthy aging, pension, reforms

## Abstract

The eligibility criteria for social pension schemes in Africa hinder equitable and healthy aging. In 2019, women in 14 sub‐Saharan African countries had an average life expectancy of 67 years but a healthy life expectancy of only 57 years, leaving them 5 years in poor health before receiving a pension at age 62. Men had a similar situation—a life expectancy of 62 years and a healthy life expectancy of 53 years, spending 10 years in poor health before becoming eligible for pensions at age 63. Many men do not receive pensions due to early death. Delays and low pension payouts contribute to a 2.5% increase in the death rate from Alzheimer's disease and dementia.

**Highlights:**

- Eligibility criteria for social pension schemes in Africa hinder equitable and healthy aging.
- Delays and low pension payouts are associated with worsening death rates from dementia.
- Average health life expectancy for both genders should serve as a basis for initiating pension payouts.

## 1 INTRODUCTION

In 1950, 6 million people in sub‐Saharan Africa were inclusive of 65.^1^ In 2017, the number jumped to 30 million; current projections are that the number will reach 226 million in 2050.^2^ However, in percentage terms, the elderly population in Africa will remain under 5% by 2050. Africa's youthful nature provides the continent with the potential to have sustainable pension systems. However, the urgency of adopting a universal pension system involving income redistribution from the vibrant youth working class to address the challenges facing the older adults cannot be overstated.^3^ With robust labor absorption in the main‐stream economy, Africa is less likely to face the worker‐to‐retiree ratio problem that the majority of (Organization for Economic Cooperation and Development (OECD) countries are currently battling in their pension systems. So far, the nominal rise in the elderly population and lack of pension coverage present challenges to African governments as they seek programs to achieve equitable healthy aging.

The World Health Organization (WHO) defines healthy aging as the process of developing and maintaining functional policies and programs that promote the well‐being of senior citizens.^4^ Regrettably, Africa is grappling with the concept of healthy aging.^5^, ^6^ The harsh reality is that working in low‐paying jobs during the stage of retirement has severe adverse effects on the health of elderly citizens.^7^ Africa is dominated by low‐paying jobs,^8^ which tragically affects the health of retiring citizens. However, there is hope. Other authors show that early pension payouts can improve the health of senior citizens by delaying the onset of physical disabilities.^9^ Therefore, the healthy aging agenda demands comprehensive pension reforms in Africa.

### 1.1 Macro‐economic determinants of healthy ageing

Global studies unequivocally establish the urgent need for social pensions as critical determinants of healthy aging.^10^ The absence of pensions often plunges disadvantaged senior citizens into poverty, rendering them unable to afford nutritious food or decent housing. These dire circumstances significantly compromise their physical and mental well‐being. In Europe, for instance, the high social welfare spending, including decent health insurance coverage and progressive pension payouts, has led to improved health outcomes for senior citizens.^11^ This compelling evidence underscores the immediate need for African governments to implement a progressive universal pension system, thereby protecting senior citizens and advancing the healthy aging agenda.

### 1.2 The landscape of African pensions

The 2022 United Nations (UN) study is a stark reminder of the pressing need for action. The UN publication reveals that a staggering 91% of sub‐Saharan African workers cannot save for their old age due to the fragility of pension systems.^12^ Therefore, the development and execution of universal pension system is significant to advance the healthy aging agenda in the region.

South Africa's success story stands as a beacon of hope, not just for its own citizens but for the entire continent. The country's robust pension system, with assets equivalent to 91% of the gross domestic product (GDP) in 2017 and serving 5.7% of the elderly population^12^ (see Table 1), is a testament to what can be achieved. This success is not an isolated case, but a shining example that significant progress is not only possible but within reach for other African nations.

**TABLE 1:** Evolution of pension assets and the elderly population in selected African countries.

	Year					
	2014	2015	2016	2017	2018	2019
Botswana (pension assets % GDP)	40.7	50.6	44.1	45.5	42.5	47.2
Botswana (% of elderly citizens 65 y+)	3.2	3.2	3.3	3.3	3.4	3.5
Botswana (% of elderly citizens 50 y+)	11.4	11.5	11.6	11.7	11.8	11.9
Botswana (% lost: 50 y+ to 65 y+)	−71.9	−72.2	−71.5	−71.8	−71.1	−70.5
Malawi (pension assets % GDP)	9.6	9.6	9.7	11.7	13.7	14.9
Malawi (% of elderly citizens 65 y+)	3.4	3.3	3.1	3.0	3.0	2.9
Malawi (% of elderly citizens 50 y+)	8.3	8.2	8.1	8.1	8.1	8.1
Malawi (% lost: 50 y+ to 65 y+)	−59.0	−59.8	−61.7	−62.9	−62.9	−64.2
Namibia (pension assets % GDP)	86.9	86.2	86.5	90.3	92.7	95.0
Namibia (% of elderly citizens 65 y+)	3.6	3.7	3.8	3.8	3.9	4.0
Namibia (% of elderly citizens 50 y+)	11.0	11.0	11.2	11.2	11.4	11.5
Namibia (% lost: 50 y+ to 65 y+)	−67.2	−66.3	−66.1	−66.1	−65.7	−65.2
South Africa (pension assets % GDP)	96.6	99.7	95.1	91.6	92.1	94.1
South Africa (% of elderly citizens 65 y+)	5.3	5.4	5.6	5.7	5.9	6.0
South Africa (% of elderly citizens 50 y+)	15.7	15.8	16.1	16.4	16.9	17.4
South Africa (% lost: 50 y+ to 65 y+)	−66.2	−65.8	−65.2	−65.2	−65.1	−65.5
Zambia (pension assets % GDP)	3.3	3.4	2.9	3.0	3.0	2.9
Zambia (% of elderly citizens 65 y+)	1.7	1.7	1.7	1.7	1.7	1.7
Zambia (% of elderly citizens 50 y+)	6.6	6.7	6.8	6.9	7.0	7.2
Zambia (% lost: 50 y+ to 65 y+)	−74.2	−74.6	−75.1	−75.4	−75.7	−76.3
Ghana (pension assets % GDP)	1.7	2.6	3.2	4.3	4.3	5.0
Ghana (% of elderly citizens 65 y+)	3.1	3.1	3.2	3.2	3.3	3.3
Ghana (% of elderly citizens 50 y+)	10.8	11.0	11.2	11.4	11.6	11.9
Ghana (% lost: 50 y+ to 65 y+)	−71.3	−71.8	−71.4	−71.9	−71.5	−72.3
Nigeria (pension assets % GDP)	5.1	5.6	6.0	6.5	6.7	7.0
Nigeria (% of elderly citizens 65 y+)	3.0	3.0	3.0	3.0	3.0	3.0
Nigeria (% of elderly citizens 50 y+)	9.7	9.7	9.7	9.7	9.7	9.8
Nigeria (% lost: 50 y+ to 65 y+)	−69.1	−69.1	−69.1	−69.1	−69.1	−69.4
Kenya (pension assets % GDP)	14.0	13.0	14.0	13.2	13.1	13.3
Kenya (% of elderly citizens 65 y+)	2.4	2.5	2.6	2.6	2.7	2.8
Kenya (% of elderly citizens 50 y+)	8.1	8.4	8.7	8.9	9.3	9.6
Nigeria (% lost: 50 y+ to 65 y+)	−70.3	−70.2	−70.1	−70.8	−71.0	−70.8
Tanzania (pension assets % GDP)	8.1	9.4	8.3	8.3	8.4	8.1
Tanzania (% of elderly citizens 65 y+)	3.1	3.1	3.1	3.1	3.1	3.1
Tanzania (% of elderly citizens 50 y+)	8.9	8.9	8.8	8.8	8.8	8.9
Tanzania (% lost: 50 y+ to 65 y+)	−65.2	−65.2	−64.8	−64.8	−64.8	−65.2

*Source*: Authors analysis based on United Nations Pension Report^12^ and World Bank Population Dataset.^14^

The South African pension system, with its diverse pension‐financing options, is built with the shared responsibility of government, employers, and employees.^13^ This collaborative effort is what makes the system so effective. The system is structured into three pillars: Pillar 1, known as non‐contributory pension (Government‐Sponsored), is designed to support the most vulnerable elderly population who have not saved for pension during their working days. This pillar ensures that the rural population is covered by a pension. Pillar 2, known as Occupational Pension Funds (Employer‐Sponsored), is a pension product of the joint efforts of employers and employees, ensuring a comfortable post‐retirement life for the working class. Pillar 3, known as Personal Pensions (Voluntary). This pillar includes private pensions, retirement annuities, and personal savings, catering to the diverse needs of individuals. Such a pillar provides a platform where even the wealthy can contribute to their retirement plans. Overall, these three pension pillars in South Africa ensure that everyone, regardless of their financial status, is covered with retirement funds. Such a pension system is engineered to advance healthy aging.

Africa should consider adopting the South African pension system as a progressive model. However, a robust means test designed to determine eligibility based on income levels and asset ownership must be implemented aggressively across the continent. This is crucial to ensure that individuals with sufficient resources and private pensions do not unfairly access the social pension, which could disadvantage the rural, non‐salaried workers.

The three‐pillar system is essential to guarantee that no one is left behind. In fact, those better‐salaried workers should invest in private pensions, as outlined in the last two pillars of the South African pension framework. The better pension payouts from private pensions will reduce the likelihood that higher earners will rely on the first pillar pension, given the more favorable returns from private pension plans. This hybrid model will bring universal pension coverage quicker to the continent.

Kenya's pension assets were equivalent to 13% of the 2017 GDP^12^ and servicing 2.6% of the elderly population.^14^ Nigeria's pension assets comprised 6.5% of the 2017 GDP,^12^ catering to 3.0% of the elderly population.^14^ Most of these pensions are highly resourced.^15^ These numbers (high pension assets as a percentage of GDP/elderly share) (see Table 1) are a testament to the resilience of pension systems in some African countries. They show that the pension systems are not just surviving but thriving. Then, where does the continent get it wrong that these pensions fail to address the healthy aging agenda?

### 1.3 Pension age eligibility and life expectancy

The gap between healthy life expectancy and pension eligibility is the crucial feature that blunts the effectiveness of African pension systems in advancing the equitable healthy aging agenda. Both men and women have bad encounters, with men having worse encounters than women. Table 2 shows the inequalities of social pension age eligibility criteria and life‐expectancy dynamics in 14 African countries with comprehensive pension system data. The pension systems in Botswana, Lesotho, Mozambique, Namibia, Eswatini, Nigeria, Kenya, Tanzania, and Uganda are structured such that men may only benefit from the pension payouts for a minuscule period (less than a year). Men's life expectancy at birth and healthy life expectancy in these nine countries are significantly lower than the pension age eligibility.^16^ This means that these men, on average, spend 12 years in poor health without pension assistance. As a result, a significant portion of them die prematurely, never having the opportunity to enjoy their pension benefits.  In most countries, delayed pension provision is associated with worsening death rates from dementia^17^ (see Table 2).

**TABLE 2:** Features of social pension, life‐expectancy matrix, and deaths from Alzheimer's diseases and dementias in Africa.

Country	Name of scheme	Year of origin	Age at pension eligibility	Targeting	2019 Healthy life expectancy (years)	2019 Life expectancy at birth (years)	Years lived in poor health before pension payout	Benefit/month (US $)	Years of enjoying pension benefits before death	% of elderly population covered	% change in death rates from Alzheimer's disease and other forms of dementia from 1980 to 2021.
Botswana	State Old Age Pension	1996	Female 65 Male 65	Universal	Female 55 Male 51	Female 65 Male 59	Female 10 Male 14	29.8	Female 0 Male 0	65	2.3
Lesotho	Old Age Pension	2004	Female 70 Male 70	Universal	Female 46 Male 42	Female 54 Male 48	Female 16 Male 22	36.7	Female 0 Male 0	61	2.2
Mauritius	Basic Retirement Pension	1950	Female 60 Male 60	Universal	Female 65 Male 62	Female 77 Male 71	Female 0 Male 0	140.5	Female 17 Male 11	100	−1.1
Mozambique	Programa de Subsídio Social Basico	1992	Female 55 Male 60	Means‐tested	Female 53 Male 48	Female 62 Male 54	Female 2 Male 12	6.6	Female 7 Male 0	24	2.3
Namibia	Old Age Pension	1948	Female 60 Male 60	Universal	Female 58 Male 53	Female 68 Male 60	Female 2 Male 7	74.6	Female 8 Male 0	100	4.1
South Africa	Old Age Grant	1927	Female 60 Male 60	Means‐tested	Female 58 Male 54	Female 68 Male 62	Female 2 Male 6	110.1	Female 8 Male 2	74	2.4
	War Veterans Grant	1928		Means‐tested				111.7			
Eswatini	Old Age Grant	2005	Female 60 Male 60	Means‐tested	Female 53 Male 47	Female 63 Male 53	Female 7 Male 13	14.4	Female 3 Male 0	77	1.9
Zambia	Social Cash Transfer Programme, Katete	2007	Female 60 Male 60	Universal	Female 56 Male 52	Female 65 Male 59	Female 4 Male 8	10.8	Female 5 Male 1	1	4.1
Cabo Verde	Pensao Social Minima	2006	Female 60 Male 60	Pension‐test	Female 67 Male 62	Female 77 Male 69	Female 0 Male 0	50.6	Female 17 Male 9	68	2.1
Nigeria	Ekiti State Social Security Scheme	2011	Female 65 Male 65	Pension‐test	Female 54 Male 53	Female 64 Male 61	Female 1 Male 4	25.1	Female 0 Male 0	<1	2.4
	Agba Osun Elderly Scheme	2012		Means‐tested				50.3			
Kenya	Older Persons Cash Transfer	2006	Female 65 Male 65	Means‐tested	Female 58 Male 56	Female 68 Male 63	Female 7 Male 9	19.4	Female 3 Male 0	15	6.6
Seychelles	Old Age Pension	1987	Female 63 Male 63	Universal	Female 66 Male 61	Female 77 Male 70	Female 0 Male 2	221.6	Female 14 Male 7	88	−0.0
Tanzania	Zanzibar Universal Pension Scheme	2016	Female 70 Male 70	Universal	Female 59 Male 57	Female 69 Male 65	Female 11 Male 13	9.2	Female 0 Male 0	<1	0.9
Uganda	Senior Citizens Grant	2011	Female 65 Male 65	Means‐tested	Female 60 Male 56	Female 70 Male 63	Female 5 Male 9	6.8	Female 5 Male 0	4	5.4
**Average**			**Female** 62 **Male** 63		**Female** 57 **Male** 53	**Female** 67 **Male** 62	**Female** 5 **Male** 10	57.3	**Female** 5 **Male** 0	48.5	2.5

*Source*: Authors analysis based on United Nations Pension Report^12^ and World Health Organization Life Expectancy Dataset.^16^ IHME dataset for Dead rates from Alzheimer's.^17^

Women's life expectancy at birth and health life expectancy are significantly better than men's life expectancy outcomes (see Table 2). This can be attributed to a variety of factors, including biological differences, lifestyle choices, and access to health care. On average, African women spend 5 years in poor health without pension assistance. For men, this figure doubles at 10 years. The early access to pensions in the female population group reinforces better life expectancy.^18^ On average, African women enjoy 5 years of pension benefits (see Table 1).  Men have zero years of pension consumption benefits, and these sharp inequalities blunt the effectiveness of these pension schemes in driving the equitable, healthy aging agenda.

Individuals with chronic health conditions are more likely to draw a pension to boost their health and physical well‐being.^19^ Therefore, correcting the age eligibility of pension payout is needed in Africa, considering the critical shortages in investments that advance mental well‐being^20^ and the lack of social welfare programs and elderly care policies.^21^ It is unfair for both gender groups to experience such delays, especially since men face higher health risks than women. This situation supports the case for providing early pensions, as many of these men may pass away before they can receive any pension benefits. The average healthy life expectancy for both genders should serve as a basis for initiating pension payouts.

### 1.4 Addressing the myth about pension reforms

Scholars who argue that pension payouts do not influence the mortality of senior citizens^22^—therefore, the modern reforms designed to enhance healthy aging are likely to fail—must be rejected. Similarly, those who advocate for increasing the pension age^23^ may undermine equitable healthy aging in Africa. Our position is that it is unsuitable for African countries to increase the pension age toward 70 years in light of the low life expectancy in this block of countries.

For example, even progressive developing countries like China reported that increasing the pension eligibility to 65 reduces labor supply,^24^ with the economy relying on the ailing labor force in poor health. Furthermore, even in high‐income countries  increasing retirement age worsens the health outcomes of senior citizens, characterized by an increasing prevalence of mental health conditions, musculoskeletal diseases, and obesity.^25^ These conditions are significant drivers for dementia onset.^26^ Other publications show that increasing retirement age toward 70 years is associated with increases in health care costs due to worsening neurological conditions.^27^ The continent may need to avoid such regressive reforms, which are contrary to the healthy aging agenda (see regressive pension outcomes in Tanzania and Lesotho in Table 2 due to the pension eligibility age set at 70 years).

### 1.5 Benefits of early pension payout

Early pension payout, initiated before poor health in Mauritius, has led to a robust improvement in life expectancy (see Table 2). Other research has consistently demonstrated that pension payouts can effectively avert depression,^28^ a significant risk factor for dementia.^26^ In South Africa, scholars have provided evidence that early pension payouts significantly reduce the risk of reported food scarcity.^29^ The literature also firmly rejects the notion that pension payouts are associated with a higher prevalence of current smoking and current alcohol consumption.^29^

Such literature informed South Africa's decision to lower the pension age eligibility criteria from 65 to 60 in 2008.^30^ This reform has yielded significant benefits, supported the healthy aging agenda, and aligned with government efforts to promote senior citizens' human rights.^21^ South Africa's successful implementation of this reform serves as a beacon for other African countries like Tanzania and Lesotho, offering a proven model for them to adopt and replicate.

### 1.6 Lessons from South Africa 2008 pension reform

Traditionally, men in South Africa were the sole recipients of social pensions at 65. However, in a departure from the general norm, the South African government introduced a 2008 pension reform,^30^ altering the age‐eligible criterion and enabling men to receive a social pension payout at 60 years—5 years earlier than most countries. This reform was a significant step toward eliminating gender disparities in pension eligibility, particularly as South African women were traditionally eligible for a social pension at 60 years of age.

The 2008 pension reform has been a key development in advancing healthy aging, as evidenced by the literature. It has not only strengthened intergenerational bonds and fostered a sense of integration among senior citizens but also significantly reduced the poverty gap ratio by 13% and increased the income of the poorest 5% of the population by a staggering 50%.^31^ These remarkable outcomes underscore the potential of such reforms to bring about positive change.

Furthermore, scholars have estimated the profound impact of the 2008 pension reform on the mental health outcomes of vulnerable men. Early pension payouts (60 years vs 65 years) led to a 3% improvement in health care utilization.^32^ The 2008 pension reform also averted depression cases, traumatic stress cases, and deaths among 60‐year‐old men by 3%, 4%, and 5%, respectively.^32^ These findings underscore the potential of early pension payouts to improve healthy aging and prevent deaths among vulnerable senior citizens.

### 1.7 Pension and dementia risk

So far, the literature shows that the prevalence of dementia is increasing in Africa.^33^ For example, the projected number of persons living with dementia in the eastern part of sub‐Saharan Africa will increase by 357% (estimated range: 323%–395%) from 2019 to 2050, and this estimate is one of the highest compared to other developing countries.^33^ Therefore, African governments are responsible for supporting healthy aging by reducing risk and preventing this anticipated increase in dementia cases.

Documented evidence reveals a beacon of hope—income can be a powerful tool to avert cognitive decline and poverty in later life, thereby reducing dementia risk.^34^ This suggests that pension payouts can be a modifiable risk factor that could potentially be harnessed to combat dementia.

Studies in Africa indicate that the rural population living below the poverty line of $1.90 per capita per day (in purchasing power parity) requires a decent income to achieve a decent standard of living, as outlined by the Sustainable Development Goals, which also include brain health.^35^ In Tanzania, the estimated decent living income is $4.04 per day, whereas in Uganda, it is $3.82 per day.^35^ This means that, on average, pension payouts in these countries should be at least $117 per month per person to promote healthy aging. However, the current average pension payout in both countries is only $8 per month (see Table 2), which is far too low to support the healthy aging agenda.

In contrast, the top three pension payouts in Africa—Seychelles, South Africa, and Mauritius—pay an average of $157 per month per person, which is sufficient to promote healthy aging.^32^ Therefore, we recommended that Africa increase its pension payouts to a minimum of $157 per month per person. The current average payout of $57.3 per month (see Table 2) is inadequate and fails to make a meaningful contribution to healthy aging, highlighting the need for reform. In fact, high pension payouts and early pension eligibility in Mauritius and Seychelles are associated with a decline in dementia death rates (see Table 2).

The negative impact of poverty and food insecurity (often prevalent among senior citizens without a pension) on cognitive function, mild cognitive impairment, and dementia risk^36^ further underscores the potential benefits of early pension payouts on healthy aging.

International examples provide compelling evidence of the potential of pension schemes to reduce dementia risk. In China and the United States, pension income was associated with better cognitive function in older adults.^37^, ^38^ This evidence was also reported in South Africa.^39^ Such evidence underscores the potential of pension reform to prevent the projected increase in dementia cases in Africa.

### 1.8 Pension and suicide rates

Despite the high pension assets relative to the percentage of the elderly population in Africa (see Table 1), the proportion of African men's lives spent in good health is low (See Table 2). The value of pension payout is low in some countries (e.g., Mozambique) (see Table 2), and indeed, the coverage is poor in countries such as Nigeria, Tanzania, and Zambia (see Table 2). These factors do not support a healthy aging agenda, and men are the biggest victims.

 Our analysis shows that, on average, from age 53, an African man spends 10 years of his life dealing with illness and disability, which ends his life at age 62. Unfortunately, the average pension system in Africa offers first payouts at age 63—when most African men are already dead.  This lack of pension support has profound implications for society, raising questions about healthy aging, justice, fairness, and human rights of older citizens.  The lack of pension support can also be linked to the high suicide rate in the African population, considering the low pension coverage and regressive social welfare policies.^40^

For example, the WHO points out that African countries have the highest suicide rates, predominantly driven by depression, anxiety, and hopelessness.^41^ Eleven people per 100,000 die by suicide in the African region. This figure is higher than the global average of 9 per 100,000 people.^41^ The male suicide rate in Africa is the highest of all world regions at 18 per 100,000, whereas the global male average is 12.2 per 100,000 people.^41^

Key to these global suicide inequalities is the lack of social protection in the African continent. For example, the 2022 report from the International Labour Organisation^42^ showed that social protection coverage rates in Europe and Central Asia covered 83.9% of the vulnerable population. This figure is 44% in Asia and the Pacific and 40% in the Arab States. Africa recorded a mere 17%, the lowest percentage globally.^43^ Therefore, it is not surprising that suicide rates are the highest in Africa, driven by the male population, which remains at the receiving end of low access to pensions despite suffering from poor health earlier (see Table 2).

Current statistics show that in old age, mental disorders represent one of the primary causes of illness and disability and are related to an increased risk of suicide.^43^, ^44^ Among the mental illnesses, depression is the leading contributor to suicide, affecting one in three adults over the inclusive of 60.^45^, ^46^ Other studies show a progressive increase in suicide rates with aging,^47^ especially among men,^48^ suggesting that the pension age eligibility criteria need serious reforms, considering that they do not serve both genders equally.

## 2 CONCLUSION

Currently, there is no internationally agreed formula to determine a reasonable pension eligibility age for pension payouts. It is crucial for African governments to develop their own pension systems, tailored to their unique demographics and health conditions, rather than benchmarking pension systems against those of wealthy nations with high life expectancy and high healthy life expectancy. The current African pension systems, prevalent in countries such as Botswana, Lesotho, Mozambique, Namibia, Eswatini, Nigeria, Kenya, Tanzania, and Uganda, are marred by significant gender inequalities for enjoying pension benefits. These disparities underscore the urgent need for reforms to promote equitable, healthy aging. The current system of delayed payouts is not only ineffective but also counterproductive to the goal of promoting equity in healthy aging. We propose that governments should consider initiating pension payouts from the onset of poor health in elderly citizens, a solution that has already been successfully implemented in Mauritius (see Table 2). This initiative will benefit the vulnerable rural populations, who often face premature death without the security of a pension. According to the World Bank,^49^ pension payouts account for ≈1% of Africa's GDP.^49^ The government might consider allocating an additional 1% to support the neglected rural population. As shown in the 2019 pension asset data in Table 1, all these countries have the capacity to afford such an expansion.

We noticed that the majority of African pension systems have a uniform retirement age for both males and females (except Mozambique) (see Table 2). However, such schemes ignore the  exposures to morbidity and mortality risks. Therefore, it is crucial for African policymakers to examine whether the health of older individuals will allow them to continue working after healthy life expectancy. This consideration is not only fair but also essential for the sustainability and effectiveness of African pension systems in advancing health aging.

Our analysis  reveals a  significant disparity in health status between males and females, which may not support increasing pension eligibility criteria. These differences also do not justify maintaining the current pension eligibility age if the continent wants to achieve equitable healthy aging. It is evident that the additional years of life after healthy life expectancy are often lived in poor health. Therefore, the majority of pension systems need to be reformed so that the payouts during these added years after healthy life expectancy can still advance healthy aging and propel life expectancy. As it stands, the payouts are too late to restore good health, especially in the male population group from countries such as Botswana, Lesotho, Mozambique, Namibia, Eswatini, Nigeria, Kenya, Tanzania, and Uganda. Amending the pension eligibility age and setting a new eligibility age between the ages of 50 and 58 is warranted, similar to pension systems in Bhuta, Burkina Faso, Congo, Figi, Guinea, Haiti, India, Indonesia, Kuwait, Malawi, Mali, Nepal, Senegal, Singapore, Solomon Islands, Sri Lanka, Thailand, Vanuatu, and Zambia.^50^ These reforms have the potential to advance healthy aging in the listed African countries. Social health insurance is another vehicle that needs to be strengthened in Africa to advance healthy aging.

## CONFLICT OF INTEREST STATEMENT

The authors declare no competing interests. Author disclosures are available in the Supporting information.

## Supporting information



Supporting Information

## References

[1] United Nations . World population prospects 2019. Accessed April 30, 2024. https://population.un.org/wpp/

[2] United Nations Department of Economic and Social Affairs, Population Division . World Population Ageing 2017 ‐ Highlights (ST/ESA/SER.A/397). 2017. Accessed April 30, 2024. https://www.un.org/en/development/desa/population/publications/pdf/ageing/WPA2017_Highlights.pdf

[3] Thovoethin P , Ewalefoh J . Universal old‐age pension: can Africa overcome its challenges?’ Afr Public Serv Deliv Perform Rev. 2018;6(1):a232. doi:10.4102/apsdpr.v6i1.232

[4] World Health Organization . World Report on Ageing and Health 2015. Accessed April 30, 2024. http://apps.who.int/iris/bitstream/10665/186463/1/9789240694811_eng.pdf?ua=1

[5] Saka S , Oosthuizen F , Nlooto M . National policies and older people's healthcare in Sub‐Saharan Africa: a scoping review. Ann Glob Health. 2019;85(1):91. doi:10.5334/aogh.2401 31251482 PMC6634477

[6] Mussie KM , Setchell J , Elger BS , Kaba M , Memirie ST , Wangmo T . Care of older persons in Eastern Africa: a scoping review of ethical issues. Front Public Health. 2022;10:923097. doi:10.3389/fpubh.2022.923097 35874990 PMC9298985

[7] Baxter S , Blank L , Cantrell A , et al. Is working in later life good for your health? A systematic review of health outcomes resulting from extended working lives. BMC Public Health. 2021;21:1356. doi:10.1186/s12889-021-11423-2 34238265 PMC8268509

[8] Kapppel R . Africa's employment challenges. Labour and Social justice Study. 2021. Accessed April 30, 2024. https://library.fes.de/pdf‐files/iez/19049.pdf

[9] Riumallo Herl C , Kabudula C , Kahn K , Tollman S , Canning D . Pension exposure and health: Evidence from a longitudinal study in South Africa. J Econ Ageing. 2022;23. doi:10.1016/j.jeoa.2022.100411 PMC9731801 36505964

[10] Scharf T , Caroline S . Inequalities in later life. Centre for Ageing Better; 2017. Accessed April 30, 2024. https://ageing‐better.org.uk/sites/default/files/2017‐12/Inequalities%20scoping%20review%20full%20report.pdf

[11] de Breij S , Huisman M , Deeg DJH . Macro‐level determinants of post‐retirement health and health inequalities: A multilevel analysis of 18 European countries. Soc Sci Med. 2020;245:112669. doi:10.1016/j.socscimed.2019.112669 31739142

[12] Nyang`oro O , Njenga G . Pension funds in sub‐Saharan Africa. WIDER Working Paper 2022/95. Accessed April 30, 2024. https://www.wider.unu.edu/sites/default/files/Publications/Working‐paper/PDF/wp2022‐95‐pension‐funds‐in‐sub‐saharan‐africa.pdf

[13] OECD . Pensions at a glance: Country profiles – South Africa. 2023. Accessed April 30, 2024. https://www.oecd.org/els/public‐pensions/PAG2021‐country‐profile‐South‐Africa.pdf

[14] World Bank Dataset . Population ages 65 and above (% of total population). Accessed April 30, 2024. https://data.worldbank.org/indicator/SP.POP.65UP.TO?view=chart

[15] Guven M . Extending pension coverage to the informal sector in Africa. Social protection and jobs discussion paper no. 1933. World Bank Group; 2019. http://documents.worldbank.org/curated/en/153021563855893271/Extending‐Pension‐Coverage‐to‐the‐Informal‐Sector‐in‐Africa

[16] World Health Organization . Life expectancy and Healthy life expectancy Data by country. Accessed April 30, 2024. https://apps.who.int/gho/data/view.main.HALEXv?lang=en

[17] IHME dataset for Dead rates from Alzheimer's,1980 to 2021 . Estimated annual number of deaths from Alzheimer's disease and other forms of dementia per 100,000 people. https://ourworldindata.org/grapher/dementia‐death‐rates?tab=table&time=earliest..2021&country=South‐East+Asia+Region+%28WHO%29~Region+of+the+Americas+%28WHO%29~Eastern+Mediterranean+Region+%28WHO%29~Western+Pacific+Region+%28WHO%29~BWA

[18] Bravo J , Ayuso M , Holzmann R , Palmer E . Addressing the life expectancy gap in pension policy. Insur Math Econ. 2021;99:200‐221.

[19] Aakvik A , Holmås H , Kjerstad E . Health status, life expectancy and early claiming of pension. SN Bus Econ. 2024;4:45. doi:10.1007/s43546-024-00640-7

[20] Mostert CM , Aballa A , Khakali L , et al. The shortfalls of mental health compartment models: a call to improve mental health investment cases in developing countries. Value Health Reg Issues. 2024;41:48‐53. doi:10.1016/j.vhri.2023.11.012 38237329

[21] Ataguba JE , Bloom DE , Scott AJ . A timely call to establish an international convention on the rights of older people. Lancet Healthy Longev. 2021;2(9):e540‐e542. doi:10.1016/S2666-7568(21)00178-1 34494015 PMC8412833

[22] Philipp J . The introduction of social pensions and elderly mortality: Evidence 1870‐1939. Ruhr. Economic Papers 808. 2019. ISBN 978‐3‐86788‐937‐7.

[23] Hagemann S , Scherger S . Increasing pension age — Inevitable or unfeasible? Analyzing the ideas underlying experts’ arguments in the UK and Germany. J Ageing Stud. 2016;39:54‐65. 10.1016/j.jaging.2016.09.004 27912855

[24] Deng Y , Fang H , Hanewald K , Wu S . Delay the pension age or adjust the pension benefit? Implications for labor supply and individual welfare in China. J Econ Beha Organ. 2023;212:1192‐1215. doi:10.1016/j.jebo.2023.06.025

[25] Barschkett M , Geyer J , Haan P , Hammerschmid A . The effects of an increase in the retirement age on health — Evidence from administrative data. J Econ Ageing. 2022;23:100403. 10.1007/s10198-022-01535-w PMC10406678 36274115

[26] Livingston G , Huntley J , Sommerlad A , et al. Dementia prevention, intervention, and care: 2020 report of the Lancet Commission. Lancet. 2020;396(10248):413‐446. doi:10.1016/S0140-6736(20)30367-6 32738937 PMC7392084

[27] Geyer J , Barschkett M , Haan P , Hammerschmid A . The effects of an increase in the retirement age on health care costs: evidence from administrative data. Eur J Health Econ. 2023;24(7):1101‐1120. doi:10.1007/s10198-022-01535-w 36274115 PMC10406678

[28] Zhou M , Sun X , Huang L . Does social pension expansion relieve depression and decrease medical costs? Evidence from the rural elderly in China. Int J Public Health. 2022;67:1604296. doi:10.3389/ijph.2022.1604296 35370536 PMC8966648

[29] Lloyd‐Sherlock P , Agrawal S , Gómez‐Olivé X . Pensions, consumption and health: evidence from rural South Africa. BMC Public Health. 2020;20:1577. doi:10.1186/s12889-020-09666-6 33081729 PMC7576783

[30] Ralston M , Schatz E , Menken J , Gómez‐Olivé FX , Tollman S . Who benefits—or does not—from South Africa's old age pension? Evidence from characteristics of rural pensioners and non‐pensioners. Int J Environ Res Public Health. 2016;13(1):85. 10.3390/ijerph13010085 PMC4730476 26712777

[31] Help Age International . Why social pensions are needed now. Help Age International Briefing on Social Pensions. 2006. Accessed April 30, 2024. http://www.globalageing.org/pension/world/2007/needed.pdf

[32] Mostert CM , Mackay D , Awiti A , Kumar M , Merali Z . Does social pension buy improved mental health and mortality outcomes for senior citizens? Evidence from South Africa's 2008 pension reform. Prev Med Rep. 2022;30:102026. doi:10.1016/j.pmedr.2022.102026 36310690 PMC9596742

[33] GBD 2019 Dementia Forecasting Collaborators . Estimation of the global prevalence of dementia in 2019 and forecasted prevalence in 2050: an analysis for the Global Burden of Disease Study 2019. Lancet Public Health. 2022;7:e105–e125. 34998485 10.1016/S2468-2667(21)00249-8 PMC8810394

[34] Samuel LJ , Szanton SL , Wolff JL , Ornstein KA , Parker LJ , Gitlin LN . Socioeconomic disparities in six‐year incident dementia in a nationally representative cohort of U.S. older adults: an examination of financial resources. BMC Geriatr. 2020;20(1):156. doi:10.1186/s12877-020-01553-4 32370792 PMC7201761

[35] Van de Ven G , de Valença A , Marinus W , et al. Living income benchmarking of rural households in low‐income countries. Food Sec.2021;13:729‐749. doi:10.1007/s12571-020-01099-8

[36] Qian H , Khadka A , Martinez SM , et al. Food insecurity, memory, and dementia among US adults aged 50 years and older. JAMA Netw Open. 2023;6(11):e2344186. doi:10.1001/jamanetworkopen.2023.44186 37988079 PMC10663972

[37] Ayyagari P , Frisvold D . The impact of social security income on cognitive function at older ages. Am J Health Econ. 2016;2(4):463‐488.

[38] Peng C , Burr JA , Han SH . Cognitive function and cognitive decline among older rural Chinese adults: the roles of social support, pension benefits, and medical insurance. Ageing Ment Health. 2023;27(4):771‐779. doi:10.1080/13607863.2022.2088693 PMC10460523 35702759

[39] Jock J , Kobayashi L , Chakraborty R , et al. Effects of pension eligibility expansion on men's cognitive function: findings from rural South Africa. J Ageing Soc Policy. 2023;1‐20. doi:10.1080/08959420.2023.2195785 PMC10533724 36975023

[40] Stewart F , Yermo J . Pensions in Africa. OECD working papers on insurance and private pensions. No. 30. OECD publishing; 2009. doi:10.1787/227444006716

[41] World Health Organization . Suicide Mortality Rate (Per 100 000 Population), WHO data. 2023. Accessed April 30, 2024. https://www.who.int/data/gho/data/indicators/indicator‐details/GHO/suicide‐mortality‐rate‐(per‐100‐000‐population

[42] International Labour Organization . World Social Protection Report 2020‐22. Accessed April 30, 2024. https://www.social‐protection.org/gimi/Media.action;jsessionid=gedX4wTENdfTov‐Xyg‐_56xcuA5yN0sX6zMXKwwZekCSBDHHLNey!‐765179005?id=18683

[43] Scott KM , Lim C , Al‐Hamzawi A , et al. Association of mental disorders with subsequent chronic physical conditions: World Mental Health Surveys From 17 Countries. JAMA Psychiatry. 2016;73:150‐158. 26719969 10.1001/jamapsychiatry.2015.2688 PMC5333921

[44] Vos T , Lim SS , Abbafati C , et al. Global burden of 369 diseases and injuries in 204 countries and territories, 1990‐2019: A systematic analysis for the Global Burden of Disease Study 2019. Lancet. 2020;396:1204‐1222. 33069326 10.1016/S0140-6736(20)30925-9 PMC7567026

[45] Hu T , Zhao X , Wu M , et al. Prevalence of depression in older adults: A systematic review and meta‐analysis. Psychiatry Res. 2022;311:114511. 35316691 10.1016/j.psychres.2022.114511

[46] Zenebe Y , Akele B , Necho M . Prevalence and determinants of depression among old age: A systematic review and meta‐analysis. Ann Gen Psychiatry. 2021;20:55. 34922595 10.1186/s12991-021-00375-x PMC8684627

[47] Alicandro G , Malvezzi M , Gallus S , La Vecchia C , Negri E Bertuccio P . Worldwide trends in suicide mortality from 1990 to 2015 with a focus on the global recession time frame. Int J Public Health. 2019;64:785‐795. 30847527 10.1007/s00038-019-01219-y

[48] Shah A , Bhat R , Zarate‐Escudero S , DeLeo D , Erlangsen A . Suicide rates in five‐year age‐bands after the age of 60 years: The international landscape. Ageing & Mental Health. 2016;20:131‐138. 10.1080/13607863.2015.1055552 26094783

[49] World Bank . Extending Pension Coverage to the Informal Sector in Africa. World Bank Discussion Paper; 2019. https://documents1.worldbank.org/curated/en/153021563855893271/pdf/Extending‐Pension‐Coverage‐to‐the‐Informal‐Sector‐in‐Africa.pdf

[50] ISSA Dataset . Pensionable ages. Country profiles. Accessed April 30, 2024. https://www.issa.int/databases/country‐profiles/pensionable‐ages

